# Early Behaviour and Biomarker Changes in ARTE10 mice reveal Translational Potential for Alzheimer’s Disease and Mood‐Related Comorbidities

**DOI:** 10.1002/alz70861_108471

**Published:** 2025-12-23

**Authors:** Ashley M Bernardo, Monika Baryla, Ewa Sokolowska, Laura Griffin, Moriah Jacobson, Guy Higgins

**Affiliations:** ^1^ Transpharmation Canada, Fergus, ON Canada; ^2^ Transpharmation Poland, Olsztyn, Warmian‐Masurian Voivodeship Poland; ^3^ Taconic Biosciences, Rensselaer, NY USA

## Abstract

**Background:**

Alzheimer’s disease (AD) is characterized by progressive cognitive decline and hallmark neuropathological changes including amyloidβ (Aβ) plaque deposition and neuroinflammation. Patients often suffer from prodromal and co‐morbid mood symptoms including anxiety and agitation which evade current therapies. The ARTE10 transgenic mouse model, which co‐expresses human APP and PS1 mutations linked to familial AD, develops early and robust Aβ pathology. Previous studies have focused primarily on cognitive changes and pathology in advanced stages of AD. Here we aimed to comprehensively characterize the ARTE10 model for prodromal and disease state behavioural phenotypes and biomarkers.

**Method:**

5‐month‐old male mice (*N* =16 WT and N=13 Homozygous ARTE10) were included in this study. The following behavioural domains were assessed with specific assays: mood: nest building, anxiety: canopy test, hyperactivity/agitation: running wheel, sensory‐motor gating: pre‐pulse inhibition, cognition: Y maze and Morris Water maze (MWM), motor function: rotarod. Plasma, CSF and brain samples were collected to evaluate the levels of NfL, amyloid and cytokines using the Meso Scale Discovery platform and then correlated to behavioural results.

**Result:**

Homozygous ARTE10 mice show significant deficits across several behavioural domains. Canopy test revealed increased anxiety‐like behaviours via reduced distance in the open zone (*p* =0.0086), and decreased looking over the edge (*p* =0.0452). Nest building demonstrated mood disturbances (*p* =0.0006), and running wheel evidenced hyperactivity (*p* =0.0233) in ARTE10 animals. Cognition working memory endpoints using Y maze did not show any significant deficits however associative learning was impacted in MWM cued learning (*p* =0.0667). Biomarker analysis revealed significantly increased Aβ42/40 ratio in the hippocampus (*p* =0.015) and cortex (*p* <0.0001) of ARTE10 mice. NfL was increased in the cortex (*p* =0.0016) and increasing in CSF (*p* =0.06). Increased TNFα (*p* =0.0039) was also found in plasma of ARTE10 animals. Correlation analysis between behaviour and biomarkers show correlations between amyloid load and some behaviours.

**Conclusion:**

The ARTE10 mouse model exhibits prodromal behavioural phenotypes related to AD, pronounced amyloid pathology and early detectable biomarkers. These features support the strong translational potential of this model to advance therapeutic developments for AD, including those related to core pathology and comorbid symptoms that are not currently managed by approved treatments.

